# Phenotypic shift from active class IV lupus nephritis to lupus podocytopathy: a case report highlighting the treatment-altering role of repeat kidney biopsy

**DOI:** 10.3389/fimmu.2026.1941706

**Published:** 2026-10-06

**Authors:** Zhifa Wang, Jinxiu Deng, Jinfeng Shen, Hui Jiang, Fuzhen Wang

**Affiliations:** Department of Nephrology, Longyan First Affiliated Hospital of Fujian Medical University, Longyan, China

**Keywords:** clinico-serological dissociation, focal segmental glomerulosclerosis, lupus nephritis, lupus podocytopathy, repeat kidney biopsy

## Abstract

**Background:**

The underlying pathophysiological mechanisms of proteinuria relapse in lupus nephritis (LN) patients during immunological remission are complex, often involving non-immune complex-mediated podocyte injury.

**Case presentation:**

We report a 21-year-old male with class IV LN and thrombotic microangiopathy (TMA). After multi-target induction and maintenance therapy, the patient achieved complete clinical remission. During subsequent follow-up, he developed progressive nephrotic-range proteinuria while maintaining serological remission (persistently negative autoantibodies and normal complement levels). Intensification of conventional immunosuppressants was ineffective. A repeat kidney biopsy revealed significant resolution of immune complex deposits but prominent focal segmental glomerulosclerosis (FSGS)-like changes with extensive podocyte foot process effacement. The regimen was adjusted to a podocyte-targeted therapy centered on tacrolimus, resulting in a rapid and sustained reduction in proteinuria.

**Conclusion:**

This case suggests that in the clinico-serological dissociation phase of LN, repeat kidney biopsy is of significant clinical value for identifying pathological phenotypic shifts, avoiding excessive immunosuppression, and guiding precision medicine.

## 1 Introduction

Lupus nephritis (LN), caused by systemic lupus erythematosus (SLE) involving the kidneys, is a major cause of end-stage kidney disease (ESKD) (1). Traditionally, LN relapse is thought to be accompanied by fluctuations in systemic immunological markers, such as elevated anti-dsDNA antibody titers and decreased complement C3 and C4 levels.

However, in clinical follow-up, some patients exhibit a “clinico-serological dissociation” phenomenon, where proteinuria recurs despite completely normal serological markers during immunological remission (2, 3).Proteinuria relapse at this stage often indicates a fundamental shift in the driving mechanisms of kidney injury. The potential mechanisms mainly include: (1) irreversible loss of nephrons due to previous severe inflammatory or vascular lesions (e.g., TMA), leading to long-term hyperfiltration and hyperperfusion in residual glomeruli, which subsequently induces hemodynamically mediated secondary FSGS (4); (2) lupus podocytopathy (LP), which is direct podocyte injury mediated by cytokines, pathologically characterized by extensive foot process effacement without obvious immune complex deposition (5–7).

Furthermore, chronic tubulointerstitial injury also participates in this pathological process. Due to the lack of histological evidence, blindly increasing the dosage of glucocorticoids or cytotoxic drugs in such patients not only fails to control non-immune-mediated podocyte injury but may also lead to severe adverse drug reactions (2, 3). Although international guidelines such as KDIGO recommend considering repeat kidney biopsy when LN treatment response is poor or the phenotype is in doubt (3, 8, 9), it is often deferred in clinical practice due to its invasiveness. This case report explores the diagnostic and therapeutic process of a patient transitioning from active class IV LN with TMA to a secondary FSGS/podocyte injury phenotype through detailed clinical and pathological data, aiming to illustrate the diagnostic and therapeutic value of repeat kidney biopsy in such clinical dilemmas.

## 2 Case presentation

### 2.1 Initial presentation (October 2020)

A 21-year-old male was admitted due to “edema of both lower limbs, facial erythema, oral ulcers, and arthralgia for 3 months.” Physical examination revealed a malar rash and pitting edema in both lower limbs.

• Laboratory tests: White blood cells 2.71×10^9^/L, hemoglobin 44 g/L, platelets 82×10^9^/L; complement C3 0.37 g/L, C4 0.08 g/L; albumin 20 g/L; serum creatinine 326 μmol/L; antinuclear antibody (ANA) 1:3200 positive; anti-dsDNA antibody 1018 IU/mL; 24-hour urine protein 1875 mg/d.
• Kidney biopsy pathology: Lupus nephritis (Class IV) combined with TMA, Activity Index (AI): 11/24, Chronicity Index (CI): 0/12.

### 2.2 Induction therapy and clinical remission (October 2020 – June 2023)

• Early intervention: In October 2020, induction therapy was initiated with prednisone 60 mg combined with hydroxychloroquine 0.2 g bid + mycophenolate mofetil 0.75 g bid. On October 19, 2020, due to an increase in serum creatinine to 467 μmol/L accompanied by oliguria, hemodialysis and plasma exchange were administered. Plasma exchange was later discontinued due to an allergic reaction. On November 10, 2020, hemodialysis was stopped after serum creatinine dropped to 208 μmol/L, and losartan 100 mg qd was used to control proteinuria.
• Multi-target therapy: In December 2020, the patient’s creatinine decreased to 144 μmol/L, but the 24-hour urine protein remained as high as 6975 mg/d. The treatment regimen was adjusted to a multi-target therapy: prednisone 60 mg combined with hydroxychloroquine 0.2 g qd + mycophenolate mofetil 0.5 g bid + tacrolimus (2 mg in the morning, 1 mg in the evening). Subsequently, the patient’s clinical manifestations improved significantly, and the dosages were gradually reduced to prednisone 10 mg qd combined with hydroxychloroquine 0.2 g qd + mycophenolate mofetil 0.25 g bid + tacrolimus 1 mg bid for maintenance therapy by March 2021.
• Maintenance and complete remission: In August 2021, the patient’s anti-dsDNA antibody was <10.0 IU/mL, and complement levels normalized. In June 2022, serum creatinine dropped to 138 μmol/L, and 24-hour urine protein decreased to 353.4 mg/d, achieving complete clinical remission (**Figure 1**). The treatment dosage was reduced to prednisone 10 mg qd combined with hydroxychloroquine 0.2 g qd + mycophenolate mofetil 0.25 g bid + tacrolimus (1 mg in the morning, 0.5 mg in the evening). In June 2023, it was further reduced to hydroxychloroquine 0.2 g qd + mycophenolate mofetil 0.25 g bid + methylprednisolone 8 mg qd.

**Figure 1:**
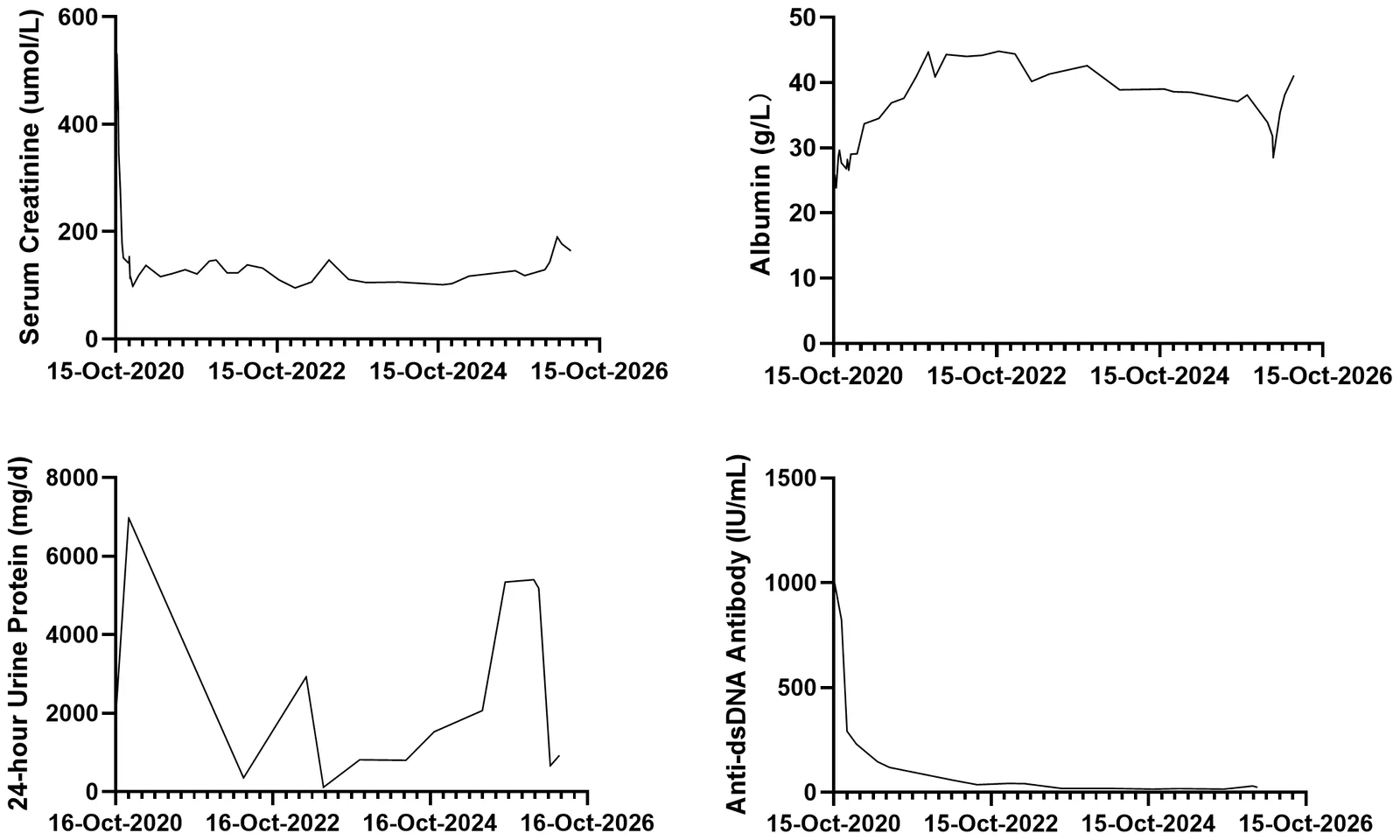
Changes of various indicators from 2020 to 2026.

Notably, during the maintenance period, the patient experienced a transient increase in proteinuria in March 2023 (24-hour urine protein reached 2928 mg/d), with a mild elevation of anti-dsDNA antibody to 41.5 IU/mL. Detailed history taking revealed an upper respiratory tract infection during this period, suggesting infection-induced transient exacerbation of proteinuria. After anti-infection treatment and rest, the 24-hour urine protein rapidly dropped to 117 mg/d in June 2023. Although there was a mild fluctuation in November 2023 (816 mg/d), immunological markers remained stable. Given that these fluctuations were considered related to reversible factors such as infection and resolved quickly on their own, a repeat kidney biopsy was not performed.

### 2.3 Proteinuria relapse and disease progression (June 2024–March 2026)

Progressive increase in proteinuria: Starting from June 24, 2024, the patient noticed increased foamy urine. Follow-up 24-hour urine protein showed a progressive increase (**Figure 1** and **Supplementary Table 1**):

• June 2024: 803 mg/d
• November 2024: 1530 mg/d
• June 2025: 2070 mg/d
• September 2025: 5340 mg/d
• February 2026: 5404 mg/d.

The immunosuppressive regimen was adjusted multiple times (**Figure 2**): prednisone 10 mg qod combined with hydroxychloroquine 0.2 g qd in June 2024; prednisone 10 mg qd combined with hydroxychloroquine 0.2 g qd + mycophenolate mofetil 0.25 g bid in November 2024; and prednisone 60 mg qd combined with hydroxychloroquine 0.2 g qd + mycophenolate mofetil 0.75 g bid in September 2025. However, proteinuria remained poorly controlled. Notably, during this stage, anti-dsDNA antibody remained persistently negative (15.9–30.1 IU/mL), complement C3/C4 were normal, immunological markers were quiescent, and ESR and hemoglobin were normal, with no signs of autoimmune hemolysis.

**Figure 2:**
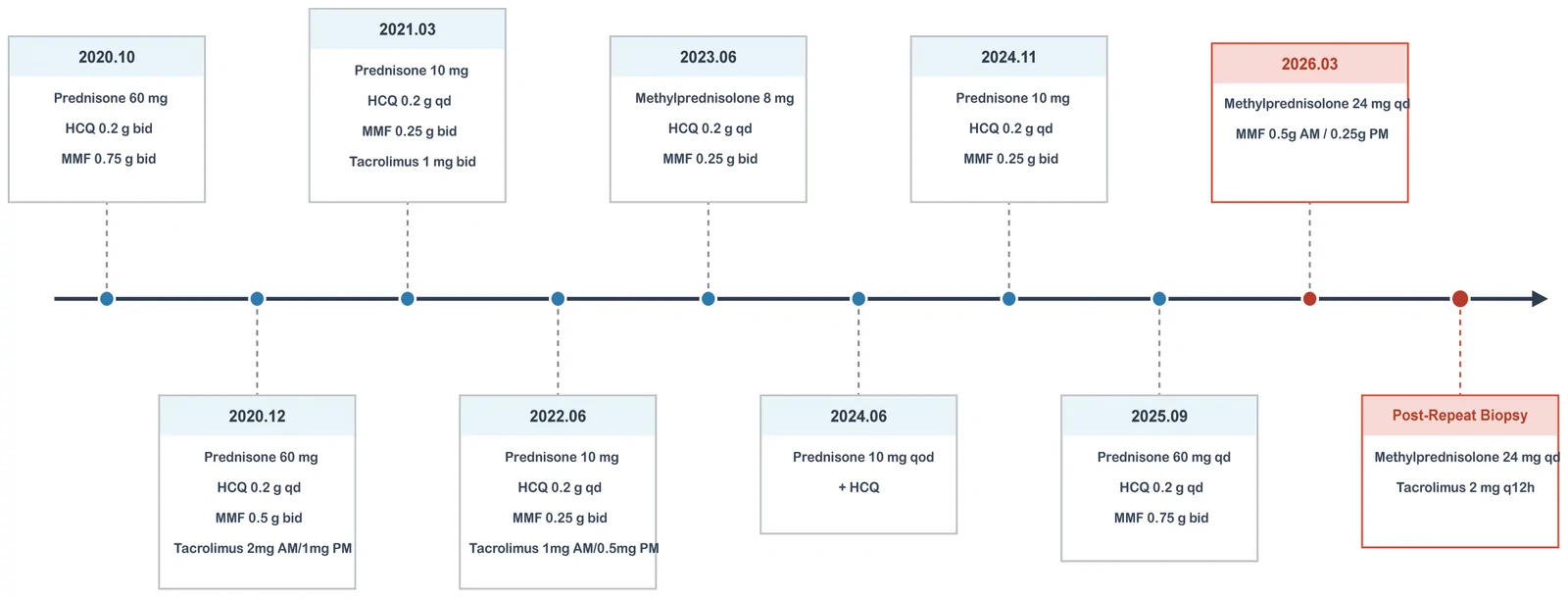
Timeline of treatment adjustments. HCQ, hydroxychloroquine; MMF, mycophenolate mofetil.

Physical examination upon admission in March 2026: BP 142/89 mmHg, no edema in lower limbs. Auxiliary tests: urine protein 4+, occult blood ±; albumin 31.8 g/L, Cr 143 μmol/L; 24-hour urine protein 5184 mg/d; anti-dsDNA antibody 24.5 IU/mL (negative). Considering the high level of proteinuria and poor treatment response, a repeat kidney biopsy was performed (**Figure 3**).

**Figure 3:**
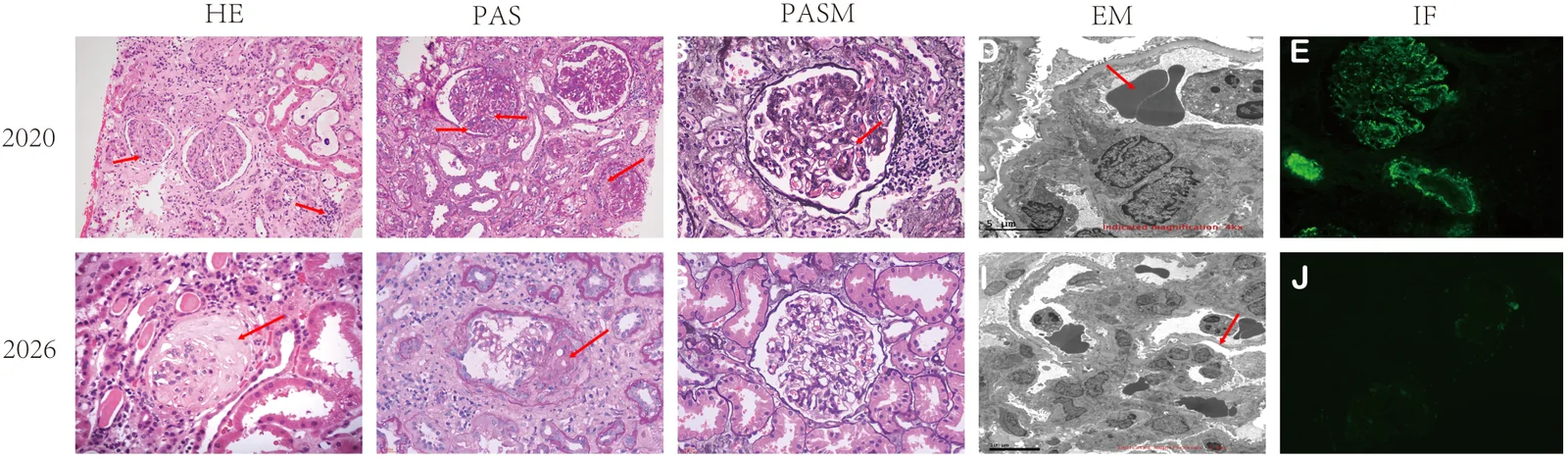
Renal pathology. **(A–E)** Initial kidney biopsy, 2020. **(F–J)** Repeat kidney biopsy, 2026. **(A)** Hematoxylin and eosin (HE) staining (400×) showing mesangial cell and matrix proliferation with segmental endothelial hyperplasia, and interstitial inflammatory cell infiltration. **(B)** Periodic acid-Schiff (PAS) staining (400×) revealing subendothelial lucent zone edema, glomerular basement membrane (GBM) thickening with “tram-track” formation, accompanied by small fibrous crescents. **(C)** Periodic acid-silver methenamine (PASM) staining (400×) demonstrating endothelial cell proliferation and “tram-track” (double contour) formation. **(D)** Electron microscopy showing mesangial, subendothelial, and sparse subepithelial electron-dense deposits, without diffuse podocyte foot process effacement. **(E)** Immunofluorescence microscopy showing strong granular deposition of C3 (3+) in the glomerular mesangium and capillary walls. **(F)** HE staining (400×) showing glomerular segmental sclerosis with fibrous crescent formation. **(G)** PAS staining (400×) showing a glomerulus with segmental sclerosis. **(H)** PASM staining (400×) showing mild lesions in non-sclerotic glomeruli without obvious proliferative changes. **(I)** Electron microscopy showing podocyte foot process effacement. **(J)** Immunofluorescence microscopy showing weakly positive C3 (1+) in the glomerulus, with minimal scattered faint green fluorescent deposits.

To rule out hereditary nephropathy, a genetic panel sequencing test for hereditary kidney diseases was completed (covering common FSGS-related genes such as NPHS1, NPHS2, WT1, ACTN4, TRPC6, INF2, etc.). The results returned negative: no pathogenic or likely pathogenic variants that could clearly explain the patient’s phenotype were detected.

### 2.4 Renal pathology findings

• Initial kidney biopsy in 2020

Light microscopy: The specimen predominantly contained renal cortex and included 17 glomeruli. No global or segmental glomerulosclerosis was observed. Two large cellular-fibrous crescents and one small cellular crescent were identified. The remaining glomeruli showed diffuse mesangial and endocapillary proliferation. Some capillary loops exhibited ischemic collapse and wrinkling, with luminal narrowing and neutrophil infiltration. Segmental thickening of the glomerular basement membrane (GBM) was present, without spike formation. Periodic acid–silver methenamine staining demonstrated focal GBM edema and double-contour formation. Fuchsinophilic deposits were observed in the mesangial and subendothelial areas; however, no typical “wire-loop” lesions were identified. No microthrombi were observed within the capillary loops.

Renal tubular epithelial cells showed granular and vacuolar degeneration. Some tubules were dilated, with epithelial flattening and loss of the brush border. Mild thickening of the tubular basement membrane was observed in some tubules, without significant tubular atrophy. The interstitium was edematous with focal inflammatory cell infiltration and no apparent fibrosis. Small arteries showed wall thickening and myxoid intimal degeneration. Some arterioles demonstrated onion-skin-like intimal hyperplasia, resulting in luminal narrowing or occlusion. Fragmented erythrocytes were observed in a small number of renal arteries, without evident fibrinoid necrosis.

Immunofluorescence: Granular deposits of IgG (2+), IgA (1+), IgM (1+), C3 (3+), and C1q (2+) were detected along the capillary loops and in the mesangial areas. C3 staining was also positive in the walls of small arteries.

Electron microscopy: Endothelial cell proliferation was observed. Lymphocytes, monocytes, and neutrophils were present within the capillary loops, some of which were compressed with narrowed lumina. No significant proliferation of parietal epithelial cells was identified. The GBM showed no obvious thickening (approximately 300–450 nm). The subendothelial space was edematous and widened. Partial podocyte foot-process effacement was present. Scattered electron-dense deposits were observed in the subendothelial, mesangial, and subepithelial regions, accompanied by mesangial cell and matrix proliferation. Tubular epithelial cells were flattened with loss of microvilli. The interstitium showed edema with lymphocytic infiltration, while arteriolar intima exhibited edema and thickening.

Pathological diagnosis: Lupus nephritis complicated by thrombotic microangiopathy (TMA).

• Repeat kidney biopsy in 2026

Light microscopy: The specimen predominantly contained renal cortex and included 18 glomeruli, of which 13 were globally sclerotic and one was segmentally sclerotic. The remaining glomeruli showed mild mesangial cell and matrix proliferation, with patent capillary loops. No GBM thickening, fuchsinophilic deposits in the mesangial, subepithelial, or subendothelial regions, mesangial interposition, or double-contour formation was observed. No proliferation of parietal epithelial cells or crescent formation was identified. Tubular epithelial cells showed vacuolar degeneration and multifocal atrophy.

Immunofluorescence: IgM (1+) and C3 (1+) staining was present, with equivocal C1q staining (±). IgG, IgA, fibrinogen, and C4d were negative.

Electron microscopy: Five glomeruli were examined, including four globally sclerotic glomeruli and one segmentally sclerotic glomerulus. In the viable glomerulus, capillary loops were patent without apparent endothelial or parietal epithelial cell proliferation. The GBM showed no significant thickening (approximately 300–400 nm), while most podocyte foot processes were effaced. No electron-dense deposits were observed in the subepithelial or intramembranous regions. Sparse electron-dense deposits were present in the mesangial area, accompanied by mild mesangial cell and matrix proliferation. Focal tubular atrophy and mild interstitial inflammatory cell infiltration were observed.

Pathological diagnosis: Focal segmental glomerulosclerosis, not otherwise specified (FSGS, NOS).

### 2.5 Clinical outcomes (March 2026–June 2026)

After the repeat kidney biopsy, the regimen was adjusted to tacrolimus 2 mg q12h + methylprednisolone tablets 24 mg qd for immunosuppressive therapy.

• Short-term efficacy (April 27, 2026): Serum creatinine 177 μmol/L, albumin 38.1 g/L, serum bicarbonate 20.7 mmol/L, potassium 4.50 mmol/L, total cholesterol 4.75 mmol/L, 24-hour urine protein 660 mg/d (an 87.2% decrease from 5184 mg/d pre-biopsy).
• Continued follow-up to June 8, 2026: Serum creatinine 164 μmol/L, albumin 41.1 g/L, urea 14.14 mmol/L, total cholesterol 6.54 mmol/L, serum bicarbonate 22.8 mmol/L, potassium 4.46 mmol/L, 24-hour urine protein 931 mg/d.

## 3 Discussion

This case vividly demonstrates the complex pathological phenotypic evolution that may occur in LN over a long-term disease course. The patient initially presented with highly active Class IV LN combined with TMA and achieved deep clinical and immunological remission after multi-target therapy. However, during the follow-up from 2024 to 2026, the patient developed progressive nephrotic-range proteinuria against a background of completely quiescent immunological markers (persistently negative anti-dsDNA antibodies and normal complement levels). This typical “clinico-serological dissociation” phenomenon suggests that the driving mechanism of kidney injury had undergone a fundamental shift and was no longer dominated by active immune complex deposition (1–3).

In the face of such proteinuria relapse, lacking histological evidence, clinicians often tend to empirically intensify conventional immunosuppressive therapy (e.g., in September 2025, prednisone was increased to 60 mg/d combined with a full dose of mycophenolate mofetil in this case). However, this strategy was ineffective and significantly increased the risk of drug toxicity (2, 3).

At this point, repeat kidney biopsy played a decisive role in guiding diagnosis and treatment (3, 8, 9). The pathological results in 2026 clearly indicated that the original active immune complex deposits (“full house” pattern, subendothelial dense deposits) had significantly resolved, replaced by extensive glomerular sclerosis and widespread effacement of podocyte foot processes in non-sclerotic glomeruli, which is highly consistent with the features of secondary FSGS or lupus podocytopathy (4, 6, 7). The potential mechanisms mainly involve two aspects: first, early severe LN and TMA led to irreversible loss of nephrons, and the compensatory hyperfiltration in residual glomeruli triggered hemodynamically mediated secondary FSGS; second, SLE-related circulating factors directly mediated podocyte cytoskeleton rearrangement and injury (5–7). Notably, to improve the differential diagnosis and rule out primary hereditary nephropathy, the patient underwent a genetic panel sequencing test for hereditary kidney diseases in March 2026 (covering common FSGS-related pathogenic genes such as NPHS1, NPHS2, WT1, ACTN4, TRPC6, INF2). The results returned negative. This molecular genetic evidence effectively ruled out the possibility of hereditary FSGS, further confirming that this case is an acquired secondary podocyte lesion/lupus podocytopathy phenotype. Furthermore, relevant viral screening was performed, effectively ruling out the possibility of secondary FSGS mediated by viral infection. It should be pointed out that although pathology showed that most glomeruli (13/18) had undergone irreversible global sclerosis, the non-sclerotic residual glomeruli must inevitably bear an extremely high compensatory hyperfiltration load. The extensive foot process effacement observed under electron microscopy occurs precisely in these residual glomeruli, which constitutes the direct source of massive proteinuria and is exactly the target for tacrolimus to exert its podocyte cytoskeleton stabilizing effect. This pathophysiological logic explains why podocyte-targeted therapy can still achieve significant proteinuria-lowering efficacy against a background of severe chronicity.

Based on precise pathological classification and genetic exclusion evidence, the treatment strategy successfully shifted from “broad-spectrum immunosuppression” to “podocyte-targeted stabilization.” In addition to its immunosuppressive effects, calcineurin inhibitors (CNIs, such as tacrolimus) can also reduce foot process effacement and constrict afferent arterioles to lower hyperfiltration by stabilizing cytoskeletal proteins such as synaptopodin within podocytes (10). After re-initiating and optimizing the tacrolimus dosage in this case, proteinuria decreased by 87.2% in the short term, and serum albumin rebounded significantly, verifying the effectiveness of this targeted treatment regimen (11, 12).

During the follow-up period, the patient experienced a transient fluctuation in serum creatinine (rising from 143 μmol/L to 177 μmol/L and then falling back to 164 μmol/L). Given that pathology already indicated approximately 40% tubular atrophy and interstitial fibrosis, this fluctuation mainly reflects the intrarenal hemodynamic changes caused by tacrolimus (afferent arteriole constriction) and the functional adaptation of the kidney during the rapid decline of proteinuria. Under close monitoring, overall renal function remained stable, and there is reason to discontinue the drug clinically.

In summary, this case indicates that in the “clinico-serological dissociation” phase of LN patients, the relapse of proteinuria is not equivalent to the relapse of lupus immune activity. Combined with molecular genetic testing to exclude genetic factors, repeat kidney biopsy has critical clinical value in accurately identifying pathological phenotypic shifts, avoiding excessive immunosuppression, and guiding individualized targeted therapy.

## Data Availability

The original contributions presented in the study are included in the article/**Supplementary Material**. Further inquiries can be directed to the corresponding authors.

## References

[1] Yu F Haas M Glassock R Zhao M-H . Redefining lupus nephritis: Clinical implications of pathophysiologic subtypes. Nat Rev Nephrol. (2017) 13:483–95. doi: 10.1038/nrneph.2017.85 28669995

[2] Parikh SV Almaani S Brodsky S Rovin BH . Update on lupus nephritis: Core curriculum 2020. Am J Kidney Dis. (2020) 76:265–81. doi: 10.1053/j.ajkd.2019.10.017 32220510

[3] Fanouriakis A Kostopoulou M Alunno A Aringer M Bajema I Boletis JN . 2019 update of the EULAR recommendations for the management of systemic lupus erythematosus. Ann Rheum Dis. (2019) 78:736–45. doi: 10.1136/annrheumdis-2019-215089 30926722

[4] De Vriese AS Sethi S Nath KA Glassock RJ Fervenza FC . Differentiating primary, genetic, and secondary FSGS in adults: A clinicopathologic approach. J Am Soc Nephrol. (2018) 29:759–74. doi: 10.1681/ASN.2017090958 29321142 PMC5827609

[5] Sakhi H Moktefi A Bouachi K Audard V Hénique C Remy P . Podocyte injury in lupus nephritis. J Clin Med. (2019) 8:1340. doi: 10.3390/jcm8091340 31470591 PMC6780135

[6] Oliva-Damaso N Payan J Oliva-Damaso E Pereda T Bomback AS . Lupus podocytopathy: An overview. Adv Chronic Kidney Dis. (2019) 26:369–75. doi: 10.1053/j.ackd.2019.08.011 31733721 PMC8405037

[7] Bomback AS Markowitz GS . Lupus podocytopathy: A distinct entity. Clin J Am Soc Nephrol. (2016) 11:547–8. doi: 10.2215/CJN.01880216 26983708 PMC4822655

[8] Parodis I Adamichou C Aydin S Gomez A Demoulin N Weinmann-Menke J . Per-protocol repeat kidney biopsy portends relapse and long-term outcome in incident cases of proliferative lupus nephritis. Rheumatology. (2020) 59:3424–34. doi: 10.1093/rheumatology/keaa129 32353879

[9] Rovin BH Adler SG Barratt J Bridoux F Burdge KA Chan TM . KDIGO 2021 clinical practice guideline for the management of glomerular diseases. Kidney Int. (2021) 100:S1–S276. doi: 10.1016/j.kint.2021.05.021 34556256

[10] Faul C Donnelly M Merscher-Gomez S Chang YH Franz S Delfgaauw J . The actin cytoskeleton of kidney podocytes is a direct target of the antiproteinuric effect of cyclosporine a. Nat Med. (2008) 14:931–8. doi: 10.1038/nm.1857 18724379 PMC4109287

[11] Kurihara I Terai C Yabe H Sugawara H . Tacrolimus induction therapy for nephrotic syndrome caused by minimal mesangial lupus nephritis with lupus podocytopathy: A case-based review. Am J Case Rep. (2022) 23:1–4. doi: 10.12659/AJCR.937201 36336892 PMC9650553

[12] Rovin BH Teng YKO Ginzler EM Arriens C Caster DJ Romero-Diaz J . Efficacy and safety of voclosporin versus placebo for lupus nephritis (AURORA 1): A double-blind, randomised, multicentre, placebo-controlled, phase 3 trial. Lancet. (2021) 397:2070–80. doi: 10.1016/S0140-6736(21)00578-X 33971155

